# Multiple functions of microsomal triglyceride transfer protein

**DOI:** 10.1186/1743-7075-9-14

**Published:** 2012-02-21

**Authors:** M Mahmood Hussain, Paul Rava, Meghan Walsh, Muhammad Rana, Jahangir Iqbal

**Affiliations:** 1 Department of Cell Biology and Pediatrics, SUNY Downstate Medical Center, Brooklyn, NY 11203, USA

**Keywords:** CD1, MTP, ApoB, Cholesterol, Triglyceride, Lipoproteins

## Abstract

Microsomal triglyceride transfer protein (MTP) was first identified as a major cellular protein capable of transferring neutral lipids between membrane vesicles. Its role as an essential chaperone for the biosynthesis of apolipoprotein B (apoB)-containing triglyceride-rich lipoproteins was established after the realization that abetalipoproteinemia patients carry mutations in the *MTTP *gene resulting in the loss of its lipid transfer activity. Now it is known that it also plays a role in the biosynthesis of CD1, glycolipid presenting molecules, as well as in the regulation of cholesterol ester biosynthesis. In this review, we will provide a historical perspective about the identification, purification and characterization of MTP, describe methods used to measure its lipid transfer activity, and discuss tissue expression and function. Finally, we will review the role MTP plays in the assembly of apoB-lipoprotein, the regulation of cholesterol ester synthesis, biosynthesis of CD1 proteins and propagation of hepatitis C virus. We will also provide a brief overview about the clinical potentials of MTP inhibition.

## Microsomal triglyceride transfer protein (MTP)

Evidence for an intracellular protein in the lumen of mammalian liver microsomes that transfers neutral lipids, triglycerides and cholesterol esters between phospholipid vesicles was first provided by Wetterau and Zilversmit [1,2]. The protein exhibits significant preference for the transfer of neutral lipids (triglycerides and cholesterol esters) compared to phospholipids. Under non-denaturing polyacrylamide gel electrophoresis conditions, the purified protein migrated as a single band [3]. However, in the presence of 0.1% SDS, two major protein bands were resolved. The P subunit (~58 kDa) was identified as the ubiquitous endoplasmic reticulum (ER) chaperone protein disulfide isomerase (PDI), whereas the larger M subunit (~97 kDa) was unique [3,4]. Therefore, MTP is a heterodimer of two distinct subunits.

## The role of PDI in MTP activity

PDI is known to facilitate proper disulfide bond formation during the biosynthesis of nascent proteins. PDI catalyzes disulfide bond formation via its isomerase and shufflase activities; both of these activities are lost when PDI associates with the M subunit. These activities, however, are recovered after disrupting the heterodimer with chaotropic agents, such as guanidine HCl, NaClO_4 _and KSCN, and non-denaturing detergents, octyl β-glucoside [4-6]. These data indicate that association of PDI with the M subunit involves non-covalent, hydrophobic interactions. This association either physically obstructs active sites present in PDI or instigates a structural change disrupting regions responsible for these activities.

PDI, by itself, lacks lipid transfer activity. Non-covalent association of the M subunit with PDI generates the fully functional lipid transfer complex, MTP. The enzymatic activities associated with PDI are unnecessary when forming an active complex. Missense mutations introduced via site-directed mutagenesis that disrupt PDI's chaperone activities have no effect on heterodimerization with the M subunit and on the formation of a fully functional lipid transfer complex [7]. Disruption of the MTP heterodimer by various agents results in the aggregation of the M subunit and loss of lipid transfer activity [6]. Thus, the role of PDI in the biosynthesis of MTP is more likely related to structural stabilization and solubilization of the complex rather than acting as an active subunit.

The manner by which the P subunit associates with the M subunit is unknown. Attempts to purify the M subunit and to recombine it with purified PDI have been unsuccessful [8]. Further, endogenous PDI present in the complex could not be exchanged *in vitro *with excess purified PDI [8]. Thus, it has been postulated that the M subunit associates with PDI during translation, although no concrete evidence is available. Once the MTP complex is formed, the two subunits do not dissociate from each other. It remains to be determined whether the complex is degraded *en bloc*, or whether it involves subunit separation followed by selective degradation of the two subunits.

## The M subunit

The M subunit belongs to a family of large lipid transfer proteins (LLTP) [9-11]. These proteins share sequence homology and have been predicted to contain similar secondary and tertiary structures. Other members of the family include apolipoprotein B (apoB), lipophorin, and vitellogenin. MTP shares extensive sequence homology with vitellogenin, an ancient protein found in vertebrates involved in the transport of lipids from extra-ovarian tissue to the oocyte. By comparison with the crystal structure of vitellogenin, MTP is predicted to have three major structural domains: N-terminal β-barrel (amino acid residues 22-297), middle α-helical (AA residues 298-603), and C-terminal β-sheet (residues 604-894) [12,13]. The N-terminal β-barrel domain mediates interaction with the N-terminus of apoB; the middle α-helical domain associates with both PDI and apoB; and the C-terminal β-sheet domain contains both the lipid binding and lipid transfer activity of MTP [12,14].

The importance of the M subunit of MTP in apoB-lipoprotein assembly was first realized by the observation that individuals with abetalipoproteinemia lack apoB-lipoproteins in their plasma and have mutations in the *MTTP *gene that result in the loss of lipid transfer activity present in the liver and intestine [15]. Abetalipoproteinemia is a rare autosomal recessive disease [15-17] characterized by a virtual absence of plasma apoB-containing lipoproteins [18]. Due to fat malabsorption and defective lipid transport, intestinal biopsies from abetalipoproteinemia patients grossly demonstrate a whitish coating and histologically visible fat-laden enterocytes. Additionally, these patients have decreased plasma triglyceride and cholesterol levels, altered membrane and lipoprotein lipid compositions, and fat soluble vitamin deficiencies (D, A, K, and E) [19]. The clinical manifestations of abetalipoproteinemia, first described by Bassen and Kornzweig, range from gastrointestinal (steatorrhea, diarrhea, failure to thrive), neurological (absent reflexes, altered sensation and movement, muscle weakness), hematological (acanthocytes, anemia, coagulopathy) to ophthalmological (pigmentary degeneration of the retina and night blindness) symptoms [19].

## Lipid transfer activities of MTP

The lipid transfer activities of MTP are measured *in vitro *using donor and acceptor vesicles [1,3]. Donor vesicles consist of unilamellar (one bilayer) phosphatidylcholine with trace amounts of radiolabeled lipids, i.e. triglycerides or cholesterol esters, and cardiolipin. After incubating donor and acceptor vesicles with a source of MTP, DEAE-cellulose (DE52) is added to remove cardiolipin containing vesicles. Acceptor vesicles in the supernatant are then counted and loss of radioactivity is used to calculate % lipid transfer per h.

Wetterau and associates also engineered donor vesicles containing fluorescent cholesteryl ester, cholesteryl 1-pyrenedecanoate, and incubated with acceptor vesicles to quantify MTP activity [6]. Following excitation at 340 nm, pyrene exhibits a complex emission spectra consisting of two peaks. The first emission is observed at 380 nm (monomer emission) and the second is observed at 470 nm (excimer or excited-state dimer emission). Time dependent reduction in excimer/monomer fluorescence ratio was correlated with the lipid transfer activity of MTP. The transformation of the excimer/monomer ratio to lipid transfer activity is not linear and consequently is neither suitable for routine MTP activity measurements nor for comparison of activity in samples of different origins. Subsequently, they reported that the monomer emission of cholesteryl 1-pyrenedecanoate at 380 nm was self-quenched when incorporated at higher concentrations into phosphatidylcholine vesicles [20] and observed that incubation of these vesicles with MTP increased fluorescence due to the binding of cholesteryl 1-pyrenedecanoate to MTP [20]. These studies were further extended to demonstrate that pyrene-labeled phospholipids and triglycerides also bind to MTP [21]. The disadvantages of the use of pyrene include the need to measure emission spectra at two different wavelengths and variability in emission spectra under different conditions and concentrations.

We described a different MTP assay that measures the transfer of nitrobenzoaxadiazol (NBD)-labeled triacylglycerols between vesicles [22]. In this assay, fluorescent lipids are quenched within the phospholipid bilayer of donor vesicles. Incubation of donor vesicles with the acceptor vesicles in the presence of different concentrations of MTP results in an increase in fluorescence with time. Once the fluorescent lipids are transferred to acceptor vesicles the fluorescence is once again quenched. This actual transfer to the acceptor vesicles can be measured after their separation by incorporating cardiolipin and dissolution with isopropanol [23]. Thus, the fluorescence increase is due to the exposure of the fluorescent lipids being transferred by MTP between donor and acceptor vesicles, and the saturation of lipid transfer represents the maximum occupancy of MTP with fluorescent lipids. This assay has been further refined to determine the transfer of cholesterol esters and phospholipids [23]. For phospholipid transfer activity, NBD-labeled phosphatidylcholine was a poor substrate as it could not be easily quenched within vesicle membranes. However, NBD-labeled phosphatidylethanolamine incorporated in phosphatidylethanolamine vesicles could be used as donor vesicles to monitor the phospholipid transfer activity of MTP. These assays are simple and amenable to throughput screening.

## MTP expression in different tissues

The M subunit expression and MTP activity is predominantly present in differentiated epithelial cells of the small intestine. MTP expression varies both longitudinally throughout the intestine and vertically within the crypt-villus axis [24-26]. Maximal expression occurs starting from the pyloric to intestinal transition in the duodenum, proximal jejunum, decreases towards the distal end and in the ileum, and is nearly absent in the colon [24]. Isolated crypts contain non-differentiated enterocytes and are devoid of both MTP mRNA and protein [26]. A graded increase in mRNA and protein levels occurs as enterocytes mature from the crypts toward the villi.

The liver is another major site of MTP expression in mammals. To date, only hepatocytes have been shown to express MTP. Within the liver, it has been reported that protein expression increases in cells proximal to the central vein and lessens toward the periphery of the lobule opposing the portal triad [27]. The physiological basis for this distribution demands further investigation. The major function of MTP in hepatocytes, as well as the previously discussed enterocytes, is to mobilize dietary and endogenous fat to other tissues through its incorporation into apoB-lipoprotein particles.

Since first described as a liver and intestine specific protein, our appreciation of MTP's tissue distribution has further evolved. Recent studies show that kidney and heart are the 3^rd ^and 4^th ^major organs expressing MTP [28]. Nevertheless, the amounts of MTP mRNA in these tissues are 3-5% of the liver levels. In the kidney, MTP is expressed in the tubular epithelial cells of the cortex but not in glomerular cells [28]. These cells also express apoB and secrete lipoproteins. It has been speculated that cortical cells might synthesize lipoproteins to recycle albumin bound fatty acids and vitamin A from the glomerular filtrate. MTP expressed in cardiac myocytes also functions in the assembly and secretion of apoB-lipoproteins [29-32]. The key purpose for lipoprotein assembly in the heart appears to be to protect this organ against the toxic accumulation of lipids. In animal models of hypoxia and diabetes, MTP activity is reduced and more lipids accumulate in the heart [32-34].

MTP is also expressed in the retina [35]. MTP protein was detected in retinal tissue as well as in a spontaneously arising transformed cell line that was shown to support secretion of apoB lipoproteins. A role similar to that illustrated for the myocardium may also be ascribed to MTP in the retina. Like heart, the retina is susceptible to lipid accumulation with the formation, as well as deposition, of cholesterol crystals. The localized deposition is associated with age-related maculopathy [35]. Further, MTP has also been reported to be expressed in neurons [35]. While speculative, it is likely to play a similar protective role that has been described in heart and retina.

It has long been recognized that MTP is expressed in the yolk sac of certain mammals [36] and is reported to be present in human placenta [37]. Cultured adipocytes, as well as fat tissue extracts from the heart and adrenal glands, also contain MTP [38,39]. Recent studies have shown that immune cells, especially lipid antigen presenting cells, contain both MTP transcripts as well as functional protein capable of transferring lipids [40,41]. Thus, MTP is more widely expressed than anticipated during early studies. MTP expression is high in cells that synthesize lipoproteins, but low in other cells. Low levels of MTP might be sufficient for the biosynthesis of CD1 proteins (see below). Higher amounts of MTP are perhaps needed for the assembly and secretion of apoB-containing lipoproteins. Tissue-specific *cis*- and *trans*-elements responsible for variable MTP expression have yet to be defined.

Recent studies point to the expression of two isoforms of MTP, only in mice, that arise from differential splicing of alternate exon 1 [39,42]. Despite two extra N terminal amino acids in the minor MTP-B isoform, both isoforms have similar lipid transfer properties. The major difference is related to their expression. The expression of the major MTP-A isoform is predominant in the liver, intestine and heart, whereas MTP-B isoform is mainly operative in macrophages and other cells that express low levels of MTP.

### Functional consequences of tissue specific ablation of MTP

Two groups have shown that MTP knockout in mice is lethal [43,44]. MTP deficiency leads to embryonic lethality predominantly between E9.5 and E10.5 [43]. Yolk sacs of MTP^-/- ^embryos contained significant amounts of lipid droplets, but lacked VLDL-size lipoproteins in their ER and Golgi compartment. Thus, a reason for embryonic lethality might be related to the inability of the yolk sac to synthesize lipoproteins for the delivery of lipids to embryos.

MTP^+/- ^mice on chow diet have half of normal mRNA, protein and activity levels. These mice have similar plasma cholesterol and triglyceride levels as MTP^+/+ ^mice, but they have lower levels (reductions of ~28%) of plasma apoB100 [43]. The livers of MTP^+/- ^mice contained higher (~20%) cholesterol and triglyceride levels and showed signs of increased lipid accumulation by Oil Red-O staining. On a high fat diet, MTP^+/- ^mice had ~20% lower levels of plasma cholesterol mainly due to reductions in VLDL/LDL. ApoB production studies showed significant reductions (~20%) in the secretion of newly synthesized apoB100 and apoB48 by isolated primary hepatocytes from MTP^+/- ^mice. Thus, both MTP alleles are required for normal plasma and hepatic lipid levels.

Chang et al. [44] and Raabe et al. [45] have floxed exons 5/6 (MTTP^fl(exon5, 6)/fl(exon5, 6)^) and exon 1 (MTTP^fl(exon1)/fl(exon1)^), respectively, of the MTP gene in mice to facilitate tissue-specific ablation. Chang et al. [44] generated liver specific MTP ablated (L-MTP^-/-^) mice by injecting adenoviruses expressing Cre-recombinase. Hepatic MTP ablation reduced plasma apoB100 and apoB48 mainly due to lower production of apoB-lipoproteins. Moreover, L-MTP^-/- ^were resistant to high-cholesterol diet induced hypercholesterolemia. Thus, hepatic MTP expression has a significant effect on plasma cholesterol levels.

Raabe et al. [45] crossed MTTP^fl(exon1)/fl(exon1) ^mice with mice expressing Cre-recombinase under the control of Mx1 promoter. The Mx1 promoter is activated after the injection of dsRNA leading to the synthesis of Cre-recombinase and deletion of MTP gene. Additionally, they deleted hepatic MTP by injecting adenoviruses expressing Cre-recombinase. Using both these approaches they achieved > 95% hepatic MTP deficiency. L-MTP^-/- ^mice had ~50% less plasma cholesterol and 30-40% less plasma triglyceride compared with floxed mice. They reported > 95% reductions in plasma apoB100 but a modest ~20% reductions in plasma apoB48. L-MTP^-/- ^hepatocytes had several lipid droplets and reduced glycogen levels. These hepatocytes did not show signs of inflammation. Electron microscopic observations showed cytosolic lipid droplets, absence of VLDL-size particles in the ER and Golgi. L-MTP^-/- ^mice were found to be more susceptible to *E. coli *lipopolysaccharide, concavalin A and *P. aeruginosa *exotoxin A induced injury [46].

Khatun et al. crossed MTTP^fl(exon1)/fl(exon1) ^mice with Albumin-Cre mice to obtain liver specific ablation of MTP [47]. MTTP gene deletion in these L-MTP^-/- ^mice reduced triglyceride transfer activity by ~80-85%. Livers of these mice had higher levels of triglyceride, cholesterol and phospholipids. Further, these mice had significantly lower levels of plasma lipids. Lipoprotein synthesis studies revealed that hepatic MTP ablation significantly reduces assembly and secretion of both apoB100 and apoB48 lipoproteins. These three L-MTP^-/- ^studies clearly establish the essential role of hepatic MTP in the production of VLDL and maintenance of plasma cholesterol and hepatic lipids.

Davidson and associates created intestine specific KO mice (I-MTP^-/-^) after crossing MTTP^fl(exon1)/fl(exon1) ^mice with mice that express Cre-recombinase under an inducible Villin promoter activated by tamoxifen [48,49]. Intestinal MTP gene deletion was associated with no weight gain and steatorrhea, gross lipid accumulation, presence of large lipid droplets in the apical portions of the enterocytes and absence of lipoproteins in the Golgi and ER [48]. MTP deficient enterocytes contained about 12-fold and 2-fold increase in triglyceride and free fatty acids, respectively [48]. Plasma lipid analysis revealed significant reductions in plasma triglyceride, cholesterol and free fatty acids due to reductions in both apoB-lipoproteins and HDL. These intestine-specific MTP deficient mice showed almost no absorption of triglycerides and 60-70% reduction in cholesterol absorption. Further, radiolabeling studies with isolated enterocytes revealed that MTP deficiency was associated with significant reductions in apoB48 secretion. These studies establish that MTP activity is essential for the assembly and secretion of apoB48-containing lipoproteins by enterocytes and that intestinal MTP contributes significantly to steady state plasma lipids.

Xie et al. [49] further examined the role of intestinal MTP in the assembly and secretion of apoB48 and apoB100 containing lipoproteins. Enterocytes only synthesized apoB48 due to efficient and complete posttranscriptional editing of the apoB mRNA by Apobec-1 enzyme [50,51]. Ablation of Apobec-1 results in the synthesis of apoB100 by the intestine [50,52]; therefore, they crossed Apobec-1^-/- ^mice with intestine-specific MTP deficient mice to obtain apoB100 expressing intestine specific MTP deficient mice (apoB100-I-Mttp^-/-^) and studied the effect of MTP deficiency on the secretion of apoB100-lipoproteins. They observed that apoB100-I-Mttp^-/- ^mice were more susceptible to death than apoB48-I-Mttp^-/- ^mice when fed high saturated or unsaturated fat but not when fed a high cholesterol diet. Investigators made two novel observations that could explain increased lethality in apoB100-I-Mttp^-/- ^mice. First, they observed that apoB100-I-Mttp^-/- ^mice did not adapt to high fat feeding by increasing the length of the small intestine as apoB48-I-Mttp^-/- ^mice did mainly due to a defect in crypt proliferation. Second, apoB100-I-Mttp^-/- ^enterocytes developed unresolved ER stress response. Although not demonstrated, the induction of the ER stress can perhaps be attributed to accumulation of apoB100 in the ER. It is known that unresolved ER stress could lead to cell death [53]. Thus, induction of unresolved ER stress might have contributed to cell death and no increase in villus length.

Iqbal et al. [54] obtain partial MTP gene deletion in the intestine by crossing MTTP^fl(exon5, 6)/fl(exon5, 6) ^mice with Villin-Cre mice. These mice had ~60-70% lower levels of intestinal MTP mRNA and activity compared with floxed mice. [^3^H]Triolein absorption studies revealed that these mice absorb ~63% less triglyceride in 2 h. These studies showed that partial ablation of MTP gene also has significant effect on acute lipid absorption.

Bjorkegren et al. [55] crossed MTTP^fl(exon1)/fl(exon1) ^mice with α-myosin heavy chain-Cre transgenic mice to generate cardiac myocytes specific ablation of the MTP gene. Heart specific MTP ablation (H-MTP^-/-^) increased triglycerides in cardiac myocytes in fasting mice. They did not report changes in MTP activity in these mice. Further, they showed that MTP inhibitors reduce secretion of apoB-containing triglyceride-rich lipoproteins by these cells. On the other hand, Bartels et al. [56] crossed MTTP^fl(exon1)/fl(exon1) ^mice expressing Cre-recombinase under the control of muscle creatine kinase promoter that is specific for skeletal and cardiac muscle cells. Surprisingly, they did not find any difference in MTP activity because the expression of MTP-A isoform was reduced by > 95% with a concomitant ~3.6-fold increase in the expression of MTP-B isoform. Both these studies, in combination with those involving altering apoB expression in the heart [29-31,55,57], support the notion that lipoprotein assembly by the heart might be to avoid cardiac lipotoxicity associated with influx of free acids during fasting or high fat feeding.

## MTP as the precursor to extracellular lipid transport systems

Lipoprotein-based lipid transport systems are also present in the plasma of egg laying animals and the hemolymph of insects. Unlike mammals, the lipid transport vehicles utilized by these organisms are not apoB-based, but instead rely on proteins with similar structure and varying capacities to carry lipids.

Insects synthesize a single multifunctional lipoprotein, lipophorin, within a specialized organ identified as the fat body [58]. Lipophorin typically consists of two apolipoproteins, apolipophorin I and apolipophorin II (~ 240 and 80 kDa, respectively). A third, apolipophorin III (18-20 kDa), may also be present to increase the overall lipid carrying capacity of the lipoprotein [59]. Lipophorin is a phospholipid rich, neutral lipid poor lipoprotein whose density is similar to mammalian HDL (~ 1.15 g/ml). The principal neutral lipid varies between triglyceride and diglyceride depending on the organism, while cholesterol ester is present in very low amounts [60]. Apolipophorin has the capacity to accept lipids via efflux from tissue (i.e. intestine) and can then deliver these to another, distant tissue without undergoing endocytosis. It therefore behaves as a continuous "lipid shuttle" that never leaves the circulation. However, some evidence does suggest that in *Locust migratoria *apolipophorin may be endocytosed similar to certain mammalian lipoproteins through an insect homolog to the mammalian low-density lipoprotein receptor [61].

Egg laying animals utilize a different lipoprotein, vitellogenin. This large apolipoprotein (~ 450 kDa) is synthesized in the liver of vertebrates and the intestines of nematodes. The major function of vitellogenin is to transport lipids to the ovary/oocytes. There it undergoes receptor-mediated endocytosis via a member of the low density-lipoprotein receptor family [62,63]. Vitellogenins, like lipophorins, are phospholipid rich, dense lipoproteins containing ~15% lipid by mass. Vitellogenins described to date contain the bulk of neutral lipids as triacylglycerols and not diacylglycerols.

Apolipophorin, vitellogenin and apoB are functionally related in that each constitutes a vehicle for extracellular lipid transport. Surprisingly, their overall relationship extends to amino acid sequence, conservation of critical cysteine residues identified to be involved in disulfide bond formation, residues required for the development of salt-bridges, as well as an overall maintenance of secondary structure (α-helical and β-sheet). These homologies provide evidence that apolipophorin, vitellogenin, and apoB are distant relatives who have undergone paralogous development [13]. As a group, they comprise the Large Lipid Transfer Protein Gene Family (LLTP) [64]. Microsomal triglyceride transfer protein is also predicted to be a member of the LLTP family based upon sequence homology [13]. Unlike other family members, MTP does not directly participate as a vehicle for transporting lipid to distant tissues. It is restricted to the intracellular compartments of the secretory pathway and is critical for the assembly of apoB and vitellogenin containing lipoproteins. It has been suggested that MTP could be the ancient protein evolved to transfer lipids [9]. The other members may have diverged to carry lipids as a cargo rather than to act as an intracellular shuttle protein for lipid transfer.

Human MTP homologues have been reported throughout a diverse collection of organisms that include mammals [65], birds [66], fish [67], insects [68], and worms [69]. While our appreciation regarding the role of MTP in mammalian apoB lipoprotein assembly and secretion is expansive, its function in organisms that do not express apoB has only recently been studied. As described for human MTP, these more ancient forms associate with PDI and localize to the secretory pathway [70,71]. Not only does this confirm the presence of MTP in distinct non-mammalian organisms, but this also suggests its mechanism for retention within the ER is preserved as well.

As has been appreciated for apoB lipoprotein assembly, recent evidence suggests that MTP may also be required for the secretion of vitellogenin [9]. When expressed by itself, vitellogenin is not secreted from a monkey kidney epithelial COS cells. However, co-expression with human MTP rescues its secretion similar to that of apoB. Further, expression of an insect (*Drosophila*) MTP ortholog in a heterologous system supported secretion of human apoB as a high-density, lipid-poor particle [68,70] and in mice as triglyceride-poor VLDL as well as phospholipid-rich HDL [47]. Surprisingly the *Drosophila *ortholog lacked triglyceride transfer activity. Later studies showed that the *Drosophila *MTP is as efficient as the human MTP in transferring phospholipids [70,71]. Thus, it appears that phospholipid transfer activity may be the ancient lipid transfer activity present in the earliest orthologs of MTP. The neutral lipid transfer activity, predominant in human MTP, might have been acquired later, during a transition from invertebrates to vertebrates [71], as the lipid rich carrier apoB evolved.

## Role of MTP in apoB-lipoprotein assembly

Nature has devised different methods to transport hydrophobic lipids in the aqueous environment of the body and tissue fluids. In the intestinal lumen, bile acid micelles solubilize dietary lipids, as well as other hydrophobic molecules, thereby facilitating their transport to enterocyte membranes for cellular uptake. Intracellularly, various proteins transport lipids to, and within, specific subcellular compartments. In the extracellular milieu, lipids are escorted between tissues by unique lipid-protein emulsions known as lipoproteins that possess an amphiphatic surface surrounding a hydrophobic core of neutral lipids, i.e. triacylglycerols and cholesterol esters. The surface layer contains a combination of phospholipids, free cholesterol, and amphiphilic proteins. In mammals, lipoproteins are synthesized predominantly in the intestine and liver. Of the various classes, apoB-lipoproteins have been of great interest owing to their enormous capacity to transport large amounts of triglycerides and their association with atherosclerosis and coronary artery disease.

The MTP's role in the assembly of apoB lipoproteins has been extensively studied and reviewed elsewhere [12,14,51,72,73]. MTP's requirement during apoB lipoprotein assembly was first demonstrated by linking abetalipoproteinemia with mutations in the M subunit [15,17]. Three independent functions, apoB-binding, membrane association, and lipid transfer, of MTP have been appreciated [12,14]. MTP was demonstrated to interact with apoB by co-immunoprecipitation, yeast two-hybrid analysis, and solid phase *in vitro *binding assays [for review, [12,14]]. These contacts were shown to be mediated through a region within the N-terminus of apoB [13,74]. Inhibition of protein-protein interactions by small molecule inhibitors decreased apoB secretion in cell culture models [75]. This suggested that not only did MTP and apoB interact within the secretory pathway, but that this interaction was necessary for the secretion of apoB lipoproteins. The lipid transfer activity associated with MTP is also vital for lipoprotein assembly and secretion. Naturally occurring missense mutations in the M subunit that result in loss of lipid transfer activity [76,77] as well as pharmacological inhibition of MTP lipid transfer activity decrease the amounts of apoB secreted [78]. Thus, both the lipid transfer and apoB-binding properties of MTP are involved in apoB-lipoprotein assembly.

Although MTP is classically identified for its ability to transfer neutral lipids, it also transfers phospholipids. Identification of a *Drosophila *ortholog of MTP that transfers phospholipids, but not triglycerides [47,68,70,71], provided additional novel insights about the need of different lipid transfer activities in apoB-lipoprotein assembly and secretion. First, *Drosophila *MTP was shown to rescue apoB secretion despite lacking triglyceride transfer activity [68]. Subsequently, we showed that in addition to rescuing apoB secretion, *Drosophila *MTP also responds to oleic acid supplementation and increases apoB secretion in cells [70]. Nonetheless, the *Drosophila *MTP was found to be ~50% as efficient as the human MTP in promoting apoB-lipoprotein assembly and secretion. These studies have now been extended to mice. Khatun et al. [47] have shown that *Drosophila *MTP can assemble apoB-lipoproteins in mouse livers. They found that *Drosophila *MTP assists in the assembly of triglyceride-poor apoB100- and apoB48-VLDL and phospholipid rich apoB48-containing HDL size particles. Hence, the phospholipid transfer activity of MTP is sufficient for the assembly and secretion of primordial lipoprotein particles. The presence of triglyceride transfer activity along with the phospholipid transfer activity, as in the human MTP, increases the number of particles assembled as well as the amounts of neutral lipids associated with them.

Though the major forms of apoB used *in vivo *for lipoprotein assembly are apoB48 and apoB100, C-terminally truncated forms of apoB have been used to decipher initial steps involved in the molecular assembly of apoB-containing lipoproteins and the role of MTP in their lipidation. The N-terminal βα1 domain (amino acids 1-731) of apoB is critical in lipoprotein assembly, as its deletion does not result in lipoprotein formation [79,80]. This N-terminal fragment has several important functional and biochemical properties. First, several disulfide bonds are present that are critical for lipoprotein assembly [81,82]. Further, this segment interacts with MTP through ionic interactions [83,84].

Secretion studies using various C-terminally truncated forms of apoB revealed that N-terminal fragments of apoB, up to apoB25 (1134 amino acids), are secreted independent of MTP [85,86]. Analyses of secreted apoB peptides showed that these peptides are associated with lipids [85-90]. Further studies have suggested that a region between B17 (771 amino acids) and B25 can associate with lipids. Based on these observations, it has been proposed that these N-terminal fragments of apoB associate with lipids in the inner leaflet of the ER and initiate the formation of "nucleation sites" on the ER membrane. Thus, initial interaction of apoB with lipids might take place independent of MTP. In the absence of the synthesis of larger hydrophobic sequences of apoB, the assembly of lipoproteins is aborted, and these peptides are secreted from cells associated with these lipids. However, when larger more hydrophobic peptides such as apoB48 and apoB100 are translated, MTP assists in bringing phospholipids and triglycerides to these peptides. In the absence of MTP these peptides are degraded by proteasomes [91,92]. Our studies indicate that the phospholipidation of these larger peptides can be accomplished by *Drosophila *MTP [47]. In the presence of additional triglyceride transfer activity, as is inherent to human MTP, lipidation and assembly of lipoproteins becomes more efficient and neutral lipid rich, buoyant lipoproteins are generated. Therefore, MTP renders naturally occurring larger apoB-peptide secretion competent by assisting their lipidation. The degree of lipidation depends on the length of the apoB-peptide, amount and type of endogenous lipids, and the inherent lipid transfer activities of MTP.

Earlier studies using MTP inhibitors indicated that secretion of apoB100 is more sensitive than apoB48 secretion [90]. Similar lesser dependence on MTP for apoB48 secretion was reported after hepatic ablation of MTP [45]. Studies of Xie et al. showed that secretion of apoB48 by enterocytes is dependent on MTP activity as intestine-specific deletion of MTP by 80% dramatically reduces apoB48 secretion by enterocytes, significantly reduces plasma lipids, and causes lipid accumulation in the intestine [48]. Secretion of both apoB100 and apoB48 from non-hepatic, non-intestinal cell lines requires MTP. Further, abetalipoproteinemia patients exhibit deficiency of both apoB48 and apoB100. Thus, it is clear that secretion of both the polypeptides require MTP. However, lesser amounts/activity of MTP might be sufficient for apoB48 lipidation/secretion, and therefore its secretion is less susceptible to MTP inhibition.

## Cholesterol ester biosynthesis

A physiological ratio of free cholesterol/phospholipids in the cellular membrane is necessary to maintain membrane fluidity and excess cellular free cholesterol is toxic to cells [93]. Hence, cellular free cholesterol levels are controlled by several pathways. One mechanism is to convert excess cellular free cholesterol and store it in ester forms. Two acyl-CoA:Cholesterol acyltransferase (ACAT) enzymes, ACAT1 and ACAT2, carry out similar cellular cholesterol esterification, but have different tissue distributions [for reviews, [94-98]]. ACAT1 is present in a variety of tissues [99-102], whereas ACAT2 is mainly expressed in enterocytes and hepatocytes [99,102-104]. Both ACAT1 and ACAT2 are integral membrane proteins with multiple transmembrane domains [105-107] and reside in the ER. They attach fatty acids from fatty acyl-CoA to the 3-hydroxyl group of membrane-associated cholesterol. Newly synthesized cholesteryl esters are first partitioned into the ER membrane, where these enzymes reside, and are later transported for storage or secretion. In the liver and intestine, cholesteryl esters are transferred to apoB-lipoproteins that are being synthesized by MTP for secretion. Therefore, inhibition/deficiency of MTP is expected to increase cellular cholesteryl esters. In contrast, Iqbal et al reported that *Mttp *gene deletion increases free cholesterol [108]. They showed that MTP inhibition or genetic ablation had no effect on ACAT1 and ACAT2 mRNA and protein levels [108]. Microsomal cholesteryl ester biosynthesis was, however, severely curtailed when MTP activity was reduced. Thus, MTP modulates cholesteryl ester synthesis by mechanisms other than transcriptional or translational control of enzymes critical for cholesterol ester biosynthesis.

To understand why MTP inhibition/deficiency leads to free cholesterol accumulation, they studied cholesterol ester biosynthesis *in vitro *using hepatic microsomes isolated from MTP deficient mice and observed significant reductions. Interestingly, supplementation of these microsomes with purified MTP restored cholesterol ester biosynthesis without affecting triglyceride and phospholipid biosynthesis. They also studied acute effects of MTP and ACAT inhibition using specific inhibitors in intestinal and hepatic cells. Individually, both inhibitors reduced cellular cholesterol esterification to similar extents and in combination they exhibited an additive effect. These studies indicated that MTP and ACAT affect different pathways in cholesterol ester biosynthesis.

To identify two different steps necessary for optimal cholesterol esterification, MTP and ACAT were expressed in cells that do not express these genes. Expression of ACAT increased cholesterol esterification; however, expression of MTP reduced cholesterol ester synthesis. Further studies showed that increased synthesis of cholesterol esters also requires apoB; expression of MTP and apoB48 in ACAT expressing cells increased cholesterol ester synthesis and secretion. Therefore, cholesterol ester synthesis and lipoprotein biogenesis act in concert to maximize cholesterol ester biosynthesis. To delineate further how these pathways coordinate in optimal cholesterol ester synthesis and to identify molecular steps involved in this process, they reconstituted cholesterol ester synthesis *in vitro *using liver microsomes, purified MTP, and LDL. Enrichment of microsomes with cholesterol esters reduced cholesterol ester biosynthesis indicating that product accumulation inhibits this process. This inhibition was avoided when purified MTP and LDL was included in the reaction mixture. These studies suggest that MTP circumvents product inhibition by removing cholesterol esters and depositing them into apoB-lipoproteins.

**Figure 1:**
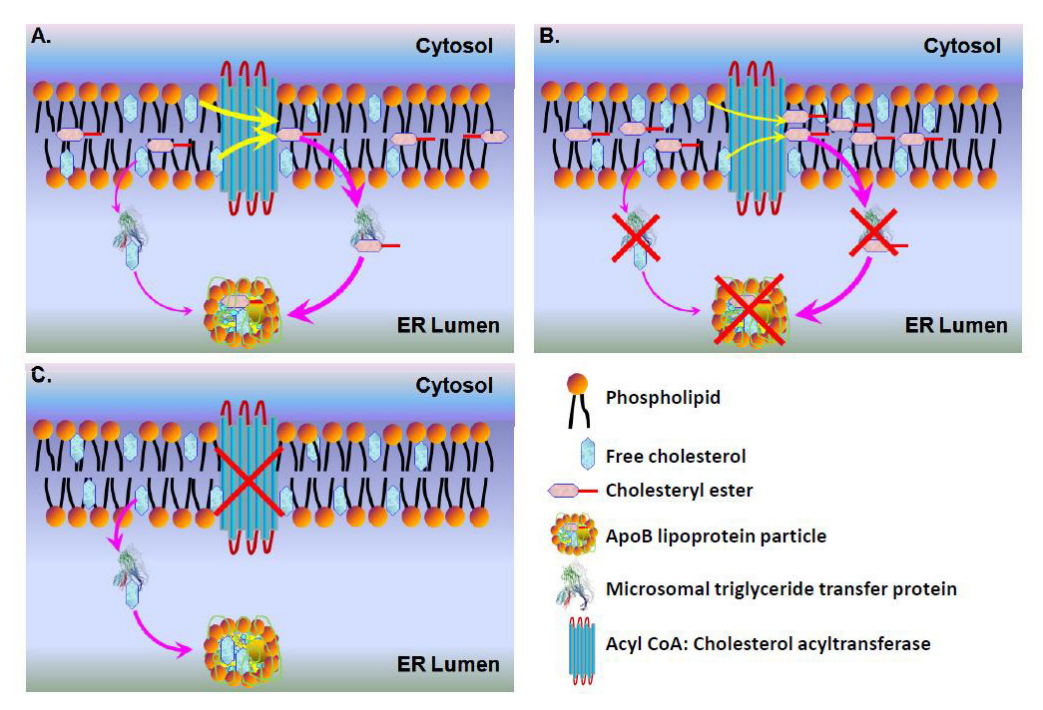
**Role of MTP in cellular cholesterol ester biosynthesis**. (**A**) ACAT, a membrane integral enzyme is shown (yellow arrows) to convert free cholesterol present in the endoplasmic reticulum (ER) leaflets into cholesterol esters that remain within the membrane bilayer. MTP is shown to transfer both free cholesterol and cholesterol esters from the ER membranes to apoB-lipoproteins in the ER lumen. It should be pointed out that MTP could transfer both free and esterified cholesterol to apoB that is still associated with membranes. The thickness of orange arrows is meant to show that MTP most likely prefers cholesteryl esters over free cholesterol for transfer. (**B**) In MTP deficient conditions, transfer of free and esterified cholesterol to apoB-lipoproteins is reduced. Initially this might lead to accumulation of cholesteryl esters. When a high enough concentration of cholesteryl esters is achieved then ACAT activity is inhibited due to product inhibition leading to accumulation of free cholesterol. (**C**) In the absence of ACAT activity, it is anticipated that cells accumulate more free cholesterol. Indeed, this is known to happen in cells that do not secrete apoB-lipoproteins, such as macrophages. However, in cells that are able to synthesize apoB-containing lipoproteins, MTP can transfer free cholesterol to lipoproteins avoiding excess free cholesterol accumulation in the ER membrane.

Based on these studies, the role of MTP in cholesterol ester biosynthesis and consequences of MTP and/or ACAT deficiency on cellular cholesterol homeostasis can be explained as follows. Under normal conditions, ACAT synthesizes cholesteryl esters and MTP transfers both free and esterified cholesterol to apoB-lipoproteins (Figure 1A). When MTP is limiting, transfer of both free cholesterol and esterified cholesterol to apoB-lipoproteins is curtailed leading to accumulation of both esterified and free cholesterol. Accumulation of esterified cholesterol inhibits esterification by ACAT enzymes contributing to further accumulation of free cholesterol (Figure 1B). Why doesn't free cholesterol accumulate in the absence of ACAT? We speculate that in lipoprotein producing cells free cholesterol in the ER membrane is removed by the MTP thereby avoiding its accumulation (Figure 1C).

## Biosynthesis of cluster of differentiation 1 (CD1) proteins

The main function of CD1 proteins is to present microbial, viral and parasite lipids to restricted populations of T lymphocytes that affect both the innate and adaptive immune response. These proteins present both endogenous and foreign lipids, glycolipids and lipopeptides, to iNKT and CD1-restricted T cells and elicit cytokine release [109-115]. These non-polymorphic molecules have been divided into 3 groups based on sequence homology. Group 1 consists of CD1a, CD1b and CD1c whereas Groups 2 and 3 contain CD1d and CD1e, respectively [109]. Humans express all three groups, but rodents only express group 2 CD1d proteins. Group 1 proteins are mainly expressed by professional antigen-presenting cells as well as thymocytes and present lipid antigens to clonally diverse T cells. In contrast, group 2 CD1d is broadly expressed in various tissues including hepatocytes and enterocytes [111] and present lipid antigens to a specialized group of T cells known as iNKT cells. CD1e has not been observed on the cell surface and, therefore, whether or not it plays a similar role in antigen presentation remains unknown [109].

CD1 molecules are transmembrane, major histocompatibility complex like glycoproteins that form heterodimers with β2-microglobulin [109,110]. They have 3 major extracellular α-helical domains. The α1 and α2 domains constitute a deep, hydrophobic, lipid binding groove while the α3-domain interacts non-covalently with β2-microglobulin [109,110,115,116]. The lipid moiety of the glycolipid and lipopeptide antigens is embedded into the hydrophobic groove, whereas the carbohydrate and peptide moieties are exposed permitting interaction with cell surface receptors present on T cells. Hence, the specificity of CD1/T cell recognition depends on cell surface CD1 molecules, their associated lipid antigens, and the repertoire of different T cell receptors.

**Figure 2:**
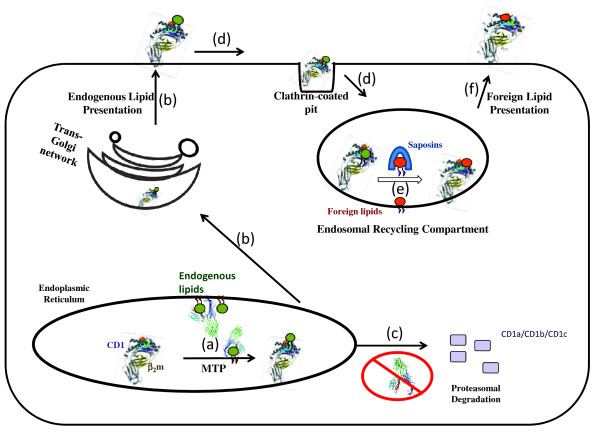
**Role of MTP in the biosynthesis of CD1 proteins:** MTP transfers endogenous lipids to newly synthesized CD1 proteins in the ER **(a)**. CD1 proteins also associate with β2-microglobulin (β2 m) before entering the secretory pathway (**b**). In the absence of MTP, group 1 CD1 proteins are subjected to proteasomal degradation (**c**). Group 2 CD1 proteins, on the other hand, are still transported to the plasma membrane in the absence of MTP. From the plasma membrane, CD1 proteins are constitutively internalized via clathrin-mediated endocytosis (**d**) and reach endosomal recycling compartment. In this compartment, saposins remove endogenous lipids and load different lipid antigens onto CD1 proteins (**e**). CD1 molecules with foreign lipids are recycled back to the plasma membrane (**f**), where they present their lipid antigens to restricted population of T cells.

CD1 molecules are synthesized with a signal sequence that directs them to the ER (Figure 2) where these proteins undergo post-translational modifications that include glycosylations, heterodimerization with β2-microglobulin and association with lipids [117]. Association with β2-microglobulin and lipids is critical for the cell surface expression of newly synthesized group 1 CD1 molecules, but not for group 2 CD1d. CD1d molecules can reach the cell surface independent of their association with either β2-microglobulin or lipids [109,110,118]. Once on the cell surface, CD1 molecules are internalized and transported to endosomes and lysosomes where endogenous lipids are exchanged with foreign lipid antigens with the help of saposins; these proteins remove endogenous lipids from CD1 molecules and then deposit a foreign lipid onto the CD1 proteins [119,120]. While the loading of foreign lipids is to stimulate an immune response, the initial association with endogenous lipids may be required for structural integrity and transport in addition to desensitizing the immune system to self-lipid antigens.

Several studies have shown that MTP plays a critical role in the loading of endogenous lipids onto newly synthesized CD1 molecules in the ER (Figure 2). Brozovic et al. [40] showed that MTP co-precipitates with CD1d from mouse hepatocytes and deletion of the *Mttp *gene increases peri-nuclear retention of CD1d while reducing their cell surface expression. A functional consequence of reduced CD1 expression is that these MTP deficient hepatocytes are less efficient in eliciting secretion of IL-2 by NKT cells in the presence of α-galactosylceramide, a ligand that occupies the hydrophobic domain on the CD1d surface. Intestinal cells also respond to α- galactosylceramide and induce secretion of IL-2 by NKT cells and MTP knockdown reduces this response. Further, oxazolone-induced colitis and associated weight loss is suppressed in MTP-deficient mice. These studies suggested that MTP transiently associates with CD1d and regulates CD1d-mediated antigen presentation in hepatic and intestinal cells.

Subsequently, evidence that MTP plays a role during CD1d antigen presentation in various antigen presenting cells was provided by Dougan et al. [41]. They showed that MTP is expressed in various antigen presenting cells, such as monocytes, B cells, splenocytes, bone marrow and monocyte derived dendritic cells and that lipid transfer activity can be measured in lysates obtained from these cells. Inhibition of MTP activity using chemical inhibitors reduced the ability of splenocytes and dendritic cells to present both endogenous and foreign antigens to NKT cells. Further, MTP knockdown in human monocyte cell line U937 also reduced antigen presentation by CD1d to NKT cells. Moreover, human B cell line C1Rd was sensitive to MTP inhibition and was reticent in presenting α- galactosylceramide to NKT cells. Thus, MTP also plays a role in antigen presentation by CD1d in a variety of antigen presenting cells.

Sagiv et al. [121] studied intracellular trafficking of newly synthesized CD1d and showed that MTP deficiency does not affect the rates of CD1d biosynthesis, glycosylation, maturation and internalization from the cell surface. However, MTP deficiency profoundly attenuated loading of antigen and recycling from lysosomes to the plasma membrane. Since MTP has not yet been shown to be present in lysosomal compartments, a likely explanation for this observation is that the consequences of MTP deficiency manifests late in the life cycle of CD1d. It is possible that CD1d molecules synthesized in the absence of MTP have structural defects in their hydrophobic groove as a consequence of deficient lipid loading during biosynthesis. Therefore, they might be unable to accept lipids in the endosomal/lysosomal compartment resulting in the degradation of these molecules rather than recycling to the cell surface with exchanged lipids.

Besides NKT cell activation, CD1d is also critical for the selection and development of NKT cells in thymocytes [42,111]. Dougan et al. [42] studied the importance of MTP in NKT cell development using fetal thymic organ cultures. Treatment of these cultures with MTP inhibitors significantly reduced the number of NKT cells that respond to endogenous and exogenous CD1d ligands. Thus, MTP is required for both selection and activation of NKT cells by CD1d.

Later, Kaser et al. [122] studied the contribution of MTP to the bioactivity of group 1 CD1 molecules. Group 1 CD1 molecules present microbial lipopeptides, glycolipids, fatty acids, phospholipids to diverse T cells that express TCR-α and TCR-β chains. They showed that MTP inhibition of monocyte derived dendritic cells led to impaired activation of CD1a, CD1b, and CD1c specific T cell activation. Further, presentation of both endogenous and foreign antigens was impaired in cells treated with MTP inhibitors. Hence, MTP also plays a role in the presentation of endogenous and foreign antigens by group 1 CD1 molecules.

As discussed before, MTP deficiency results in abetalipoproteinemia characterized by the absence of apoB-containing lipoproteins. Zeissig et al. [123] studied the presentation of self and foreign antigens by different CD1 molecules with dendritic cells isolated from abetalipoproteinemia patients. They showed that monocyte derived dendritic cells were unable to stimulate T cells in co-cultures when incubated in the presence or absence of exogenous antigens. Therefore, the presentation of both self and foreign antigens is defective in monocyte-derived dendritic cells obtained from abetalipoproteinemia patients. As discussed before, selection of thymic iNKT cells requires CD1d and MTP. They also measured iNKT cells and found that abetalipoproteinemia patients had low to undetectable levels of iNKT cells in their plasma. These studies indicate that MTP deficiency in humans significantly reduces CD1d immune-mediated cytokine response by reducing the number of iNKT cells as well as their response to cells bearing an antigen-protein complex.

They also studied the amounts of CD1 molecules present on the cell surface in abetalipoproteinemia dendritic cells. Cell surface expression of group 1 molecules was significantly reduced in abetalipoproteinemia patients. Expression of group 1 CD1 molecules could be increased when cells were incubated with proteasomal inhibitors indicating that in the absence of MTP these molecules undergo proteasomal degradation similar to that shown for apoB. Despite reductions in iNKT cell activation, cell surface expression of group 2 CD1d was not diminished in abetalipoproteinemia monocyte-derived dendritic cells. Further, incubation of these cells with proteasomal inhibitors did not increase CD1d cell surface expression. This is in contrast to observations made in mice hepatocytes [40] suggesting for possible cell specific differences. Therefore, it appears that biosynthesis and cell surface expression of group 1 CD1 proteins is more dependent on MTP than the cell surface expression of CD1d.

The studies summarized above indicate that MTP is critical for the bioactivity of CD1 molecules. To understand how MTP plays a role in the biosynthesis of CD1 molecules, an *in vitro *assay [41] was developed based on solid-liquid interphase apoB-MTP binding assays [74,83,84,124] to assess whether MTP can transfer lipids to CD1d. Purified CD1d was immobilized and incubated with lipid vesicles that contained NBD-phosphatidylethanolamine in the presence and absence of purified MTP. MTP was able to transfer NBD-phosphatidylethanolamine to CD1d but not to bovine serum albumin or major histocompatibility complex 1 [41,42]. In contrast, MTP was unable to transfer NBD-triacylglycerol to CD1d. These studies indicated that MTP facilitates the transfer of phospholipids from membranes to CD1d.

In short, it can be concluded that MTP plays a role in the proper lipidation of CD1 proteins in the ER. After this lipidation CD1 proteins are directed to the cell surface and then undergo endocytosis (Figure 2). In the endosomal/lysosomal compartment, endogenous lipids are exchanged for a different endogenous or foreign lipid by saposins [119,120] and recycled back to cell surface. When occupied with an antigenic glycolipid or lipopeptide, CD1 proteins interact with NKT cells (in the case of group 2) or clonally diverse T cells (in the case of group 1) to elicit an inflammatory response. In the absence of MTP, group 1 CD1 molecules are perhaps degraded by ER associated proteasomal degradation. In contrast, CD1d biosynthesis and cell surface trafficking are less severely altered when MTP is deficient. However, after internalization, its recycling to the cell surface is impaired most likely because it is unable to acquire lipids necessary for this event to transpire.

## MTP and Hepatitis C virus

Hepatitis C virus (HCV) is the leading cause of chronic viral hepatitis. Patients who develop the disease have an increased risk of hepatic steatosis, cirrhosis, and hepatocellular carcinoma. HCV particles circulate as lipo-viral particles which are rich in triglycerides, but also contain apolipoprotein B, HCV RNA, and core proteins [125-127]. The presence of HCV with apoB-lipoproteins prompted studies to ask whether lipoprotein assembly or MTP plays a role in viral propagation. Several studies have now established that lipoprotein assembly and MTP activity is required for viral production. For example, inhibition of lipid transfer activity of MTP or siRNA mediated knock down of apoB inhibits HCV secretion [128-131]. But lipoprotein assembly and MTP activity is not required for viral replication. Thus, viral replication takes place independent of lipoprotein assembly, and nascent viral particles get incorporated into the core of lipoproteins prior to secretion.

Apart from the role of MTP in HCV propagation, experiments have been performed to study the effect of HCV on MTP. Using transgenic mice, Perlemuter et al. [132] showed that over expression of HCV core protein interferes with VLDL assembly and secretion. The overexpression of viral core protein inhibited the lipid transfer activity associated with MTP without affecting its protein levels, or those of PDI, in mouse liver. This led the investigators to conclude that HCV core protein inhibits MTP lipid transfer activity causing hepatic steatosis. Subsequent to these studies, Domitrovic et al. [130] demonstrated that HCV non-structural proteins decrease apoB secretion and MTP activity using a subgenomic replicon system in Huh-7 cells. They further showed that reduction in MTP activity was due to a decrease in MTP promoter activity and subsequent transcription. It now appears that two mechanisms may exist for viral-induced MTP inhibition. The first works by decreasing transcription, while the other uses a still undefined means to inhibit MTP lipid transfer activity. Future studies focusing on *cis *and *trans *factors responsible for this response will undoubtedly further our understanding and medical treatment of this viral infection.

Thus, these studies indicate that although the virus hijacks lipoprotein assembly for its secretion, high concentrations of virus inhibit lipoprotein assembly. There is no good explanation to rationalize these two incompatible observations. It is possible that at early stages of infection the virus hijacks lipoproteins for its propagation and transmission to other tissues [127]. However, after reaching a higher titer, the virus might try to undergo dormancy to avoid immune detection by slowing down its propagation.

## MTP as a therapeutic target

MTP has been a favorite target to lower plasma lipids and treat disorders characterized by higher production of apoB-containing lipoproteins such as atherosclerosis, metabolic syndrome, familial combined hyperlipidemia, homozygous and heterozygous familial hypercholesterolemia and hypertriglyceridemia [73,133-140]. Now recognizing that MTP is also involved in the immune response against foreign lipid antigens, targeting MTP might also be useful for modulating the inflammatory response during T cell mediated processes such as inflammatory bowel disease, autoimmune hepatitis and asthma [141]. Several MTP antagonists have been identified that decrease lipoprotein production and plasma lipids [73,78,136,138,142]. Following promising preclinical studies, some of these drugs were evaluated in humans [140,142]. In a placebo-controlled, double-blind dose escalation study, Implitapide (Bay-13-9952) has been shown to reduce plasma total cholesterol, LDL cholesterol, apoB and triglyceride with no significant elevations in plasma transaminases [143]. A similar study in healthy volunteers, CP-346086 significantly reduced plasma total cholesterol, LDL cholesterol, and apoB [138]. Cuchel et al. [144] performed dose escalation studies using Lomitapide (BMS-201308 or AEGR-733) in familial hypercholesterolemia patients with promising reductions in plasma cholesterol and apoB levels. Samaha et al. [145] performed dose escalation studies with Lomitapide in moderate hypercholesterolemic patients and again found significant reductions in total cholesterol, LDL cholesterol and apoB. These studies provide proof of concept that MTP inhibition can be an effective therapy to lower plasma cholesterol and apoB. However, these studies showed that 10-30% of the individuals treated with MTP inhibitors experience increased plasma levels of liver transaminases and greater hepatic lipid content [144,145]. Thus, there is a need for novel methods to inhibit MTP without increasing hepatic lipids and plasma transaminases. Otherwise, MTP therapy might be limited to certain situations where alternative therapeutic interventions to lower plasma lipids do not exist such as familial hypercholesterolemia where the alternative treatments (i.e. liver transplant) are associated with high risks.

To avoid hepatic lipid accumulation and plasma transaminase elevation, it has been proposed that intestine-specific MTP inhibition might be beneficial because of the inherent property of the intestine to self-renew. In fact, intestine-specific MTP inhibitor, JTT-130, has been shown to lower plasma triglyceride and LDL cholesterol in guinea pigs without increasing hepatic triglyceride [146]. Similarly, another intestine-specific compound, SLx-4090, has been shown to lower plasma lipids [137]. Thus, an intestine-specific inhibition of MTP might avoid hepatic toxicity. However, as discussed before intestinal MTP deficiency in mice and humans is associated with gastrointestinal disturbances such as steatorrhea, diarrhea, flatulence, nausea, impulse to defecate, increased stool frequency, vomiting, heartburn and stomach pain. Although, these disturbances can be avoided by administering MTP inhibitors 4 h after the meal [142], long-term consequences related to intestine-specific MTP inhibition need careful evaluation before promoting this approach.

Other strategies to garner the full potentials of MTP therapy can also be explored. It might be possible to capitalize on natural compounds that have been shown to inhibit MTP activity, such as flavanoids (naringenin from grapefruits and hesperitin from oranges) [147], isoflavones (genestein and daidzein from soya beans) [148] and garlic extracts [149] for therapeutic benefits. Another possibility is to specifically inhibit only one of the lipid transfer activities of MTP. MTP can transfer neutral lipids and phospholipids. Since phospholipid has been shown to be sufficient for lipoproteins assembly [47,70], it might be possible to inhibit its triglyceride transfer activity to lower plasma lipids. Yet another possibility is to adapt a combined therapy involving agents that avoid hepatic lipid accumulation along with MTP antagonists.

## Future directions

We have discussed the role of MTP in apoB-lipoprotein assembly, cholesterol ester biosynthesis, and CD1 protein synthesis. Although significant information has been garnered about MTP structure and activity, very little is known about its biosynthesis and degradation. Similarly, mechanisms controlling different levels of MTP expression in different tissues and cells have not been explained. Furthermore, the function of MTP in different tissues and cells that do not express apoB and CD1 proteins, such as adipocytes, needs to be elucidated. We also discussed the emerging relationship between HCV and MTP. It is likely that MTP might also be involved in many yet unidentified biochemical, cellular and physiological processes.

Because of its role in apoB-lipoprotein assembly, MTP has been targeted to reduce plasma lipids and their atherosclerotic potential. However, these drug therapies are associated with significant and worrisome changes in hepatophysiology [133,144]. If safe drugs to target MTP become available, their efficacy in the treatment of HCV can be evaluated. Since CD1d-mediated antigen presentation contributes to various immune responses associated with infection, allergy, cancer etc., MTP inhibitors might also be useful in reducing severity of these responses.

Significant investments have been made in the identification and evaluation of MTP inhibitors. But, much less time and money has been spent on explaining the molecular basis for adverse events associated with MTP therapy. For example, very little is known about the types of lipids accumulating in the liver after MTP inhibition and their role in the release of liver-specific transaminases. Because of its role in cholesterol ester biosynthesis, it is possible that adverse events associated with MTP inhibition might be related to free cholesterol accumulation. More studies are needed to establish a cause and relationship between the types of lipids that accumulate during MTP deficiency and their role in the release of liver enzymes. Several approaches to avoiding adverse events associated with MTP inhibition have been laid out above that can be experimentally evaluated.

An area that needs more investigation relates to adverse events associated with intestinal MTP inhibition. Molecular basis for diarrhea and other intestinal complications are poorly explained. Another aspect of MTP therapy that has garnered little attention is its effect on heart and kidney functions. Although effects on these tissues may not be significant during acute treatments, they could be potentially consequential in long-term therapy.

## Abbreviations

ACTA: Acyl-CoA:Cholesterol Acyltransferase; ApoB: apolipoprotein B; CD1: cluster of differentiation 1; ER: endoplasmic reticulum; MTP: microsomal triglyceride transfer protein; NBD: nitrobenzoaxadiazol; NK: natural killer; PDI: protein disulfide isomerase.

## Competing interests

The authors declare that they have no competing interests. This work was partially supported by NIH grants DK-46900 and HL-95924 MMH.

## Authors' contributions

MMH conceived the idea and wrote the manuscript. PR wrote sections (MTP expression in different tissues and MTP as the precursor to extracellular lipid transport systems), critically read and edited the final manuscript. MW furnished a draft on "Biosynthesis of CD1 proteins", critically read/edited and made Figure 2. MR provided a draft on 'MTP and Hepatitis C Virus". JI made Figure 1. All authors read and approved the final manuscript.
